# ASSOCIATION BETWEEN GERIATRIC HOME VISIT AND EMERGENCY ROOM OR HOSPITAL UTILIZATION: A RETROSPECTIVE STUDY

**DOI:** 10.1093/geroni/igz038.3474

**Published:** 2019-11-08

**Authors:** Xin Zhang, Paul Takahashi

**Affiliations:** Mayo Clinic, Rochester, Minnesota, United States

## Abstract

Homebound older adults tend to have more medical comorbidities, higher risk of mortality, and higher healthcare utilization compared to non-homebound, community dwelling older adults in the U.S. Preventative primary care home visit have been shown to reduce hospitalizations and total healthcare costs in this population. Through a retrospective study, we aim to explore characteristics associated with ED and hospital utilization in patients who have received a home visit. A total 608 subjects, 70% female and median age of 86 years, were involved. A 184 (30%) of homebound subjects were hospitalized or visited the ED in 90 days. Comparing those with hospitalization or ED and those without, there was no significant difference in sex, age, race, marital status, or advance care planning. A 74% of those with 90 day ED/hospitalization had prior ED visit within 1 year of home visit compared to 59% of those without (p=0.0004). In addition, 58% of those with 90 day ED/hospitalization had prior hospitalization within 1 year of home visit compared to 44% of those without (p=0.0015). They also had significantly higher number of total medical comorbidities (median 4.5 vs 4, p=0.02). Our study suggests that prior healthcare utilization and medical comorbidity burden may be better predictors of 90 day hospitalization or ED use in geriatric patients who have received a home visit. Advance care planning and age did not significantly differ between the two groups in our study. Further studies should be performed to validate our findings in a prospective manner.

